# A monoclinic polymorph of (1*E*,5*E*)-1,5-bis­(2-hy­droxy­benzyl­idene)thio­carbono­hydrazide

**DOI:** 10.1107/S1600536811030340

**Published:** 2011-07-30

**Authors:** Bonell Schmitt, Thomas Gerber, Eric Hosten, Richard Betz

**Affiliations:** a Nelson Mandela Metropolitan University, Summerstrand Campus, Department of Chemistry, University Way, Summerstrand, PO Box 77000, Port Elizabeth 6031, South Africa

## Abstract

The title compound, C_15_H_14_N_4_O_2_S, is a derivative of thio­ureadihydrazide. In contrast to the previously reported polymorph (ortho­rhom­bic, space group *Pbca*, *Z* = 8), the current study revealed monoclinic symmetry (space group *P*2_1_/*n*, *Z* = 4). The mol­ecule shows non-crystallographic *C*_2_ as well as approximate *C*_s_ symmetry. Intra­molecular bifurcated O—H⋯(N,S) hydrogen bonds, are present. In the crystal, inter­molecular N—H⋯S hydrogen bonds and C—H⋯π contacts connect the mol­ecules into undulating chains along the *b* axis. The shortest centroid–centroid distance between two aromatic systems is 4.5285 (12) Å.

## Related literature

For the crystal structure of the ortho­rhom­bic polymorph of the title compound reported without three-dimensional coordinates, see: Yanping *et al.* (1999 ▶). For the crystal structure of a methyl­ated derivative of the title compound, see: Affan *et al.* (2010 ▶). For graph-set analysis of hydrogen bonds, see: Etter *et al.* (1990 ▶); Bernstein *et al.* (1995 ▶). Structures containing similar C=S distances were retrieved from the Cambridge Structural Database (Allen, 2002 ▶). For chelate ligands in coordination chemistry, see: Gade (1998 ▶).
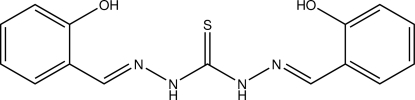


## Experimental

### 

#### Crystal data

C_15_H_14_N_4_O_2_S
*M* *_r_* = 314.36
Monoclinic, 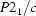

*a* = 5.6020 (1) Å
*b* = 7.4260 (2) Å
*c* = 34.5220 (8) Å
β = 91.225 (1)°
*V* = 1435.80 (6) Å^3^
*Z* = 4
Mo *K*α radiation
μ = 0.24 mm^−1^
*T* = 200 K
0.20 × 0.17 × 0.10 mm

#### Data collection

Bruker APEXII CCD diffractometer
Absorption correction: multi-scan (*SADABS*; Bruker, 2008 ▶) *T*_min_ = 0.879, *T*_max_ = 1.000
13304 measured reflections
3578 independent reflections
2830 reflections with *I* > 2σ(*I*)
*R*_int_ = 0.027

#### Refinement

*R*[*F* ^2^ > 2σ(*F* ^2^)] = 0.048
*wR*(*F* ^2^) = 0.116
*S* = 1.11
3578 reflections
209 parameters
H atoms treated by a mixture of independent and constrained refinement
Δρ_max_ = 0.31 e Å^−3^
Δρ_min_ = −0.30 e Å^−3^

### 

Data collection: *APEX2* (Bruker, 2010 ▶); cell refinement: *SAINT* (Bruker, 2010 ▶); data reduction: *SAINT*; program(s) used to solve structure: *SIR97* (Altomare *et al.*, 1999 ▶); program(s) used to refine structure: *SHELXL97* (Sheldrick, 2008 ▶); molecular graphics: *ORTEP-3* (Farrugia, 1997 ▶) and *Mercury* (Macrae *et al.*, 2008 ▶); software used to prepare material for publication: *SHELXL97* and *PLATON* (Spek, 2009 ▶).

## Supplementary Material

Crystal structure: contains datablock(s) I, global. DOI: 10.1107/S1600536811030340/kj2182sup1.cif

Supplementary material file. DOI: 10.1107/S1600536811030340/kj2182Isup2.cdx

Structure factors: contains datablock(s) I. DOI: 10.1107/S1600536811030340/kj2182Isup3.hkl

Supplementary material file. DOI: 10.1107/S1600536811030340/kj2182Isup4.cml

Additional supplementary materials:  crystallographic information; 3D view; checkCIF report

## Figures and Tables

**Table 1:** Hydrogen-bond geometry (Å, °) *C*_g_1 and *C*_g_2 are the centroids of the C11–C16 and C21–C26 rings, respectively.

*D*—H⋯*A*	*D*—H	H⋯*A*	*D*⋯*A*	*D*—H⋯*A*
O1—H81⋯N2	0.84	1.87	2.597 (2)	144
O1—H81⋯S1	0.84	2.99	3.7096 (14)	145
O2—H82⋯N4	0.84	1.89	2.617 (2)	144
O2—H82⋯S1	0.84	3.08	3.8135 (16)	147
N1—H71⋯S1^i^	0.86 (2)	2.53 (2)	3.3514 (17)	159 (2)
N3—H73⋯S1^i^	0.85 (3)	2.82 (3)	3.5605 (18)	147 (2)
C16—H16⋯*C*_g_2^i^	0.95	2.81	3.423 (2)	123
C26—H26⋯*C*_g_1^i^	0.95	2.74	3.438 (2)	130

Symmetry code: (i) 
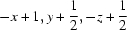
.

**Fig. 1.**
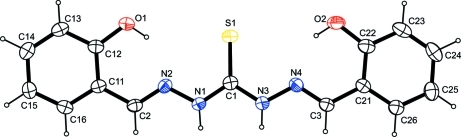
The molecular structure of the title compound, with atom labels and anisotropic displacement ellipsoids (drawn at 50% probability level).

**Fig. 2.**
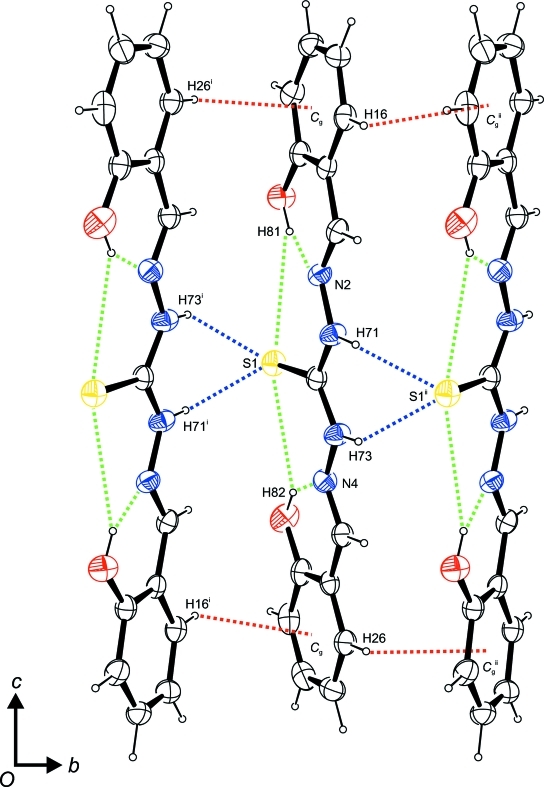
Intermolecular contacts, viewed along [-1 0 0]. Depicted are intramolecular (green dashed lines) as well as intermolecular (blue dashed lines) hydrogen bonds and C–H···π contacts (red dashed lines). Symmetry operators: i -x + 1, y - 1/2, -z + 1/2; ii -x + 1, y + 1/2, -z + 1/2.

## supplementary crystallographic information

### Comment

Chelate ligands have found widespread use in coordination chemistry due to the enhanced thermodynamic stability of resultant coordination compounds in relation to coordination compounds exclusively applying comparable monodentate ligands (Gade, 1998). Combining different donor atoms, a molecular set-up to accomodate a large variety of metal centers of variable Lewis acidity is at hand. In this aspect, the title compound seemed particularily interesting due to its use as strictly neutral or – depending on the pH value – as anionic or cationic ligand. In addition, due to the set-up of its donor atoms, a multitude of differently-sized chelate ligands can be formed. The presence of a thioketo group as well as amino groups, hydroxyl groups and imine-type nitrogen atoms further enhances the versatility of the title compound's ligating abilities. In our continuous interest in elucidating the rules influencing the formation of coordination compounds with different set-ups of *NOS*-donor atoms, we determined the crystal structure of the title compound to enable comparative studies with geometric parameters in envisioned coordination compounds. Although the compound has been reported to crystallize in the orthorhombic space group *Pbca* (Yanping *et al.*, 1999), we found a monoclinic polymorph. Furthermore, no three-dimensional coordinates have been deposited for the former structure solution. The molecular and crystal structure of a methyl-substituted derivative of the title compound is apparent in the literature as well (Affan *et al.*, 2010).

The molecule is essentially planar. The least-squares planes defined by the carbon atoms of the phenyl groups (including the respective C=N moiety) intersect at an angle of only 5.33 (8) °. The least-squares plane defined by the atoms of the central N_2_C=S motif encloses angles of 7.26 (8) ° and 11.75 (7) ° with the aforementioned least-squares planes, respectively (Fig. 1). The C=N double bonds are invariably (*E*)-configured. The length of the C=S bond is in good agreement with values reported for other thioketones whose crystal structural data have been deposited with the Cambridge Structural Database (Allen, 2002), the reported range being 1.297–1.864 Å.

In the crystal structure, intra- as well as intermolecular hydrogen bonds are apparent. While the intramolecular hydrogen bond – stemming from the hydroxyl group – shows bifurcation between the sulfur atom as well as the imine-type nitrogen atom, the intermolecular hydrogen bonds exclusively have the sulfur atom as acceptor. The presence of the sulfur-supported hydrogen bond is complemented by the results of IR spectroscopy that show the presence of three bands in the region for hydrogen bonds between oxygen, nitrogen and sulfur. In addition, C–H···π contacts can be observed that involve hydrogen atoms on the aromatic system. In terms of graph-set analysis (Etter *et al.*, 1990; Bernstein *et al.*, 1995), the descriptor for the bifurcated hydrogen bond is *S*(6)*S*(9) on the unitary level while the amino-group-supported hydrogen bonds necessitate a *C*^1^_1_(4)*C*^1^_1_(4) descriptor on the same level. A binary descriptor of *R*^1^_2_(6) emphasizes the "chelation" of the sulfur atom by the two secondary amino groups. In total, the molecules are connected to waved, zigzag-type chains along the crystallographic *b* axis. The shortest intercentroid distance between two π-systems was measured at 4.5285 (12) Å and involves both aromatic moieties (Fig. 2).

### Experimental

The compound was prepared upon reacting thiocarbohydrazide (0.50 mmol) with *ortho*-hydroxybenzaldehyde (1.00 mmol) in refluxing ethanol (15 ml) under nitrogen in analogy to a published procedure (Yanping *et al.*, 1999). Crystals suitable for the X-ray diffraction study were obtained upon slow evaporation of the reaction mixture.

### Refinement

Carbon-bound H atoms were placed in calculated positions (C—H 0.95 Å) and were included in the refinement in the riding model approximation, with *U*(H) set to 1.2*U*_eq_(C). The H atom of the hydroxyl groups were allowed to rotate with a fixed angle around their respective C—O bonds to best fit the experimental electron density (HFIX 147 in the *SHELX* program suite (Sheldrick, 2008)). The H atoms of the amine groups were located on a difference Fourier map and refined with individual displacement parameters.

### Crystal data

**Table:**

C_15_H_14_N_4_O_2_S	*F*(000) = 656
*M**_r_* = 314.36	*D*_x_ = 1.454 Mg m^−^^3^
Monoclinic, *P*2_1_/*c*	Mo *K*α radiation, λ = 0.71069 Å
Hall symbol: -P 2ybc	Cell parameters from 3781 reflections
*a* = 5.6020 (1) Å	θ = 3.3–28.2°
*b* = 7.4260 (2) Å	µ = 0.24 mm^−^^1^
*c* = 34.5220 (8) Å	*T* = 200 K
β = 91.225 (1)°	Block, colourless
*V* = 1435.80 (6) Å^3^	0.20 × 0.17 × 0.10 mm
*Z* = 4	

### Data collection

**Table:**

Bruker APEXII CCD diffractometer	3578 independent reflections
Radiation source: fine-focus sealed tube	2830 reflections with *I* > 2σ(*I*)
graphite	*R*_int_ = 0.027
φ and ω scans	θ_max_ = 28.3°, θ_min_ = 2.4°
Absorption correction: multi-scan (*SADABS*; Bruker, 2008)	*h* = −7→7
*T*_min_ = 0.879, *T*_max_ = 1.000	*k* = −9→9
13304 measured reflections	*l* = −46→45

### Refinement

**Table:**

Refinement on *F*^2^	Primary atom site location: structure-invariant direct methods
Least-squares matrix: full	Secondary atom site location: difference Fourier map
*R*[*F*^2^ > 2σ(*F*^2^)] = 0.048	Hydrogen site location: inferred from neighbouring sites
*wR*(*F*^2^) = 0.116	H atoms treated by a mixture of independent and constrained refinement
*S* = 1.11	*w* = 1/[σ^2^(*F*_o_^2^) + (0.0459*P*)^2^ + 0.6828*P*] where *P* = (*F*_o_^2^ + 2*F*_c_^2^)/3
3578 reflections	(Δ/σ)_max_ < 0.001
209 parameters	Δρ_max_ = 0.31 e Å^−^^3^
0 restraints	Δρ_min_ = −0.30 e Å^−^^3^

### Fractional atomic coordinates and isotropic or equivalent isotropic displacement parameters (Å^2^)

**Table:**

	*x*	*y*	*z*	*U*_iso_*/*U*_eq_	
S1	0.12373 (8)	0.21558 (7)	0.258163 (14)	0.02903 (14)	
O1	0.1741 (2)	0.2387 (2)	0.36537 (4)	0.0324 (3)	
H81	0.2203	0.2637	0.3430	0.049*	
O2	−0.1378 (2)	0.2458 (2)	0.15547 (4)	0.0383 (4)	
H82	−0.0382	0.2723	0.1731	0.057*	
N1	0.5251 (3)	0.3922 (2)	0.27723 (4)	0.0281 (4)	
H71	0.646 (4)	0.457 (3)	0.2711 (6)	0.037 (6)*	
N2	0.4749 (3)	0.3542 (2)	0.31484 (4)	0.0265 (3)	
N3	0.4199 (3)	0.4051 (2)	0.21391 (5)	0.0294 (4)	
H73	0.546 (5)	0.466 (4)	0.2103 (7)	0.053 (8)*	
N4	0.2684 (3)	0.3694 (2)	0.18331 (4)	0.0274 (3)	
C1	0.3648 (3)	0.3442 (2)	0.24941 (5)	0.0248 (4)	
C2	0.6209 (3)	0.4048 (2)	0.34167 (5)	0.0256 (4)	
H2	0.7674	0.4613	0.3355	0.031*	
C3	0.3279 (3)	0.4210 (2)	0.14954 (5)	0.0255 (4)	
H3	0.4739	0.4834	0.1460	0.031*	
C11	0.5583 (3)	0.3740 (2)	0.38171 (5)	0.0237 (4)	
C12	0.3391 (3)	0.2956 (3)	0.39193 (5)	0.0258 (4)	
C13	0.2860 (3)	0.2726 (3)	0.43090 (6)	0.0303 (4)	
H13	0.1391	0.2186	0.4378	0.036*	
C14	0.4458 (4)	0.3279 (3)	0.45949 (6)	0.0319 (4)	
H14	0.4073	0.3126	0.4860	0.038*	
C15	0.6625 (4)	0.4057 (3)	0.44992 (6)	0.0312 (4)	
H15	0.7718	0.4439	0.4697	0.037*	
C16	0.7166 (3)	0.4266 (3)	0.41139 (6)	0.0270 (4)	
H16	0.8656	0.4783	0.4049	0.032*	
C21	0.1696 (3)	0.3832 (2)	0.11659 (5)	0.0239 (4)	
C22	−0.0524 (3)	0.2973 (3)	0.12065 (6)	0.0287 (4)	
C23	−0.1919 (4)	0.2586 (3)	0.08796 (6)	0.0345 (5)	
H23	−0.3413	0.2000	0.0907	0.041*	
C24	−0.1148 (4)	0.3048 (3)	0.05168 (6)	0.0368 (5)	
H24	−0.2114	0.2772	0.0295	0.044*	
C25	0.1036 (4)	0.3915 (3)	0.04711 (6)	0.0336 (5)	
H25	0.1562	0.4234	0.0221	0.040*	
C26	0.2414 (3)	0.4301 (3)	0.07948 (5)	0.0277 (4)	
H26	0.3896	0.4903	0.0765	0.033*	

### Atomic displacement parameters (Å^2^)

**Table:**

	*U*^11^	*U*^22^	*U*^33^	*U*^12^	*U*^13^	*U*^23^
S1	0.0237 (2)	0.0299 (3)	0.0336 (3)	−0.0028 (2)	0.00372 (17)	0.0010 (2)
O1	0.0251 (7)	0.0398 (8)	0.0323 (7)	−0.0086 (6)	0.0007 (5)	0.0014 (6)
O2	0.0254 (7)	0.0448 (9)	0.0447 (8)	−0.0075 (6)	0.0017 (6)	0.0091 (7)
N1	0.0250 (8)	0.0338 (9)	0.0255 (8)	−0.0060 (7)	0.0014 (6)	0.0048 (7)
N2	0.0276 (8)	0.0275 (8)	0.0245 (8)	0.0005 (7)	0.0027 (6)	0.0037 (6)
N3	0.0279 (8)	0.0351 (10)	0.0250 (8)	−0.0074 (7)	−0.0010 (6)	0.0033 (7)
N4	0.0258 (8)	0.0285 (9)	0.0277 (8)	−0.0019 (7)	−0.0019 (6)	−0.0007 (7)
C1	0.0240 (9)	0.0211 (9)	0.0292 (9)	0.0036 (7)	0.0019 (7)	0.0003 (7)
C2	0.0230 (9)	0.0236 (9)	0.0303 (9)	−0.0002 (7)	0.0023 (7)	0.0041 (7)
C3	0.0235 (9)	0.0235 (9)	0.0296 (9)	−0.0008 (7)	−0.0003 (7)	−0.0003 (7)
C11	0.0233 (8)	0.0193 (9)	0.0286 (9)	0.0021 (7)	0.0031 (7)	0.0026 (7)
C12	0.0226 (8)	0.0226 (9)	0.0322 (9)	0.0013 (7)	0.0016 (7)	0.0008 (8)
C13	0.0265 (9)	0.0299 (10)	0.0347 (10)	0.0021 (8)	0.0073 (8)	0.0050 (8)
C14	0.0372 (11)	0.0314 (11)	0.0273 (9)	0.0063 (9)	0.0054 (8)	0.0023 (8)
C15	0.0358 (10)	0.0312 (11)	0.0264 (9)	0.0010 (9)	−0.0037 (8)	−0.0011 (8)
C16	0.0234 (9)	0.0235 (9)	0.0340 (10)	−0.0007 (7)	−0.0018 (7)	0.0009 (7)
C21	0.0224 (8)	0.0197 (9)	0.0296 (9)	0.0029 (7)	−0.0016 (7)	−0.0014 (7)
C22	0.0230 (8)	0.0233 (9)	0.0397 (10)	0.0028 (8)	−0.0002 (7)	0.0021 (8)
C23	0.0250 (9)	0.0249 (10)	0.0534 (13)	0.0002 (8)	−0.0081 (9)	−0.0013 (9)
C24	0.0354 (11)	0.0314 (11)	0.0428 (11)	0.0076 (9)	−0.0158 (9)	−0.0074 (9)
C25	0.0367 (11)	0.0353 (12)	0.0288 (10)	0.0099 (9)	−0.0027 (8)	−0.0024 (8)
C26	0.0254 (9)	0.0255 (10)	0.0319 (10)	0.0032 (8)	−0.0002 (8)	0.0008 (8)

### Geometric parameters (Å, °)

**Table:**

S1—C1	1.6867 (19)	C12—C13	1.394 (3)
O1—C12	1.356 (2)	C13—C14	1.381 (3)
O1—H81	0.8400	C13—H13	0.9500
O2—C22	1.359 (2)	C14—C15	1.390 (3)
O2—H82	0.8400	C14—H14	0.9500
N1—C1	1.349 (2)	C15—C16	1.379 (3)
N1—N2	1.364 (2)	C15—H15	0.9500
N1—H71	0.86 (2)	C16—H16	0.9500
N2—C2	1.279 (2)	C21—C26	1.395 (3)
N3—C1	1.348 (2)	C21—C22	1.407 (3)
N3—N4	1.367 (2)	C22—C23	1.389 (3)
N3—H73	0.85 (3)	C23—C24	1.377 (3)
N4—C3	1.278 (2)	C23—H23	0.9500
C2—C11	1.451 (2)	C24—C25	1.394 (3)
C2—H2	0.9500	C24—H24	0.9500
C3—C21	1.455 (2)	C25—C26	1.375 (3)
C3—H3	0.9500	C25—H25	0.9500
C11—C16	1.396 (3)	C26—H26	0.9500
C11—C12	1.411 (2)		
			
C12—O1—H81	109.5	C13—C14—C15	120.65 (17)
C22—O2—H82	109.5	C13—C14—H14	119.7
C1—N1—N2	118.38 (15)	C15—C14—H14	119.7
C1—N1—H71	119.2 (15)	C16—C15—C14	119.13 (18)
N2—N1—H71	121.9 (15)	C16—C15—H15	120.4
C2—N2—N1	119.14 (16)	C14—C15—H15	120.4
C1—N3—N4	119.14 (16)	C15—C16—C11	121.80 (18)
C1—N3—H73	121.2 (17)	C15—C16—H16	119.1
N4—N3—H73	119.7 (17)	C11—C16—H16	119.1
C3—N4—N3	118.46 (16)	C26—C21—C22	118.51 (17)
N3—C1—N1	113.38 (16)	C26—C21—C3	119.15 (16)
N3—C1—S1	123.54 (14)	C22—C21—C3	122.33 (17)
N1—C1—S1	123.06 (14)	O2—C22—C23	117.24 (17)
N2—C2—C11	118.67 (16)	O2—C22—C21	123.01 (17)
N2—C2—H2	120.7	C23—C22—C21	119.73 (18)
C11—C2—H2	120.7	C24—C23—C22	120.37 (19)
N4—C3—C21	119.25 (17)	C24—C23—H23	119.8
N4—C3—H3	120.4	C22—C23—H23	119.8
C21—C3—H3	120.4	C23—C24—C25	120.72 (19)
C16—C11—C12	118.32 (17)	C23—C24—H24	119.6
C16—C11—C2	119.49 (16)	C25—C24—H24	119.6
C12—C11—C2	122.18 (17)	C26—C25—C24	118.90 (19)
O1—C12—C13	117.24 (16)	C26—C25—H25	120.6
O1—C12—C11	122.97 (16)	C24—C25—H25	120.6
C13—C12—C11	119.79 (17)	C25—C26—C21	121.76 (18)
C14—C13—C12	120.30 (18)	C25—C26—H26	119.1
C14—C13—H13	119.9	C21—C26—H26	119.1
C12—C13—H13	119.9		
			
C1—N1—N2—C2	−177.46 (17)	C13—C14—C15—C16	−0.2 (3)
C1—N3—N4—C3	−176.94 (18)	C14—C15—C16—C11	0.8 (3)
N4—N3—C1—N1	−178.39 (16)	C12—C11—C16—C15	−0.7 (3)
N4—N3—C1—S1	3.2 (3)	C2—C11—C16—C15	177.92 (17)
N2—N1—C1—N3	173.21 (16)	N4—C3—C21—C26	−176.19 (17)
N2—N1—C1—S1	−8.4 (2)	N4—C3—C21—C22	2.4 (3)
N1—N2—C2—C11	176.08 (16)	C26—C21—C22—O2	179.74 (17)
N3—N4—C3—C21	179.64 (16)	C3—C21—C22—O2	1.1 (3)
N2—C2—C11—C16	179.37 (17)	C26—C21—C22—C23	1.1 (3)
N2—C2—C11—C12	−2.0 (3)	C3—C21—C22—C23	−177.55 (17)
C16—C11—C12—O1	−179.45 (17)	O2—C22—C23—C24	−179.10 (18)
C2—C11—C12—O1	1.9 (3)	C21—C22—C23—C24	−0.4 (3)
C16—C11—C12—C13	0.0 (3)	C22—C23—C24—C25	−0.2 (3)
C2—C11—C12—C13	−178.65 (17)	C23—C24—C25—C26	0.1 (3)
O1—C12—C13—C14	−179.87 (17)	C24—C25—C26—C21	0.6 (3)
C11—C12—C13—C14	0.7 (3)	C22—C21—C26—C25	−1.2 (3)
C12—C13—C14—C15	−0.6 (3)	C3—C21—C26—C25	177.45 (17)

### Hydrogen-bond geometry (Å, °)

**Table:**

*C*_g_1 and *C*_g_2 are the centroids of the C11–C16 and C21–C26 rings, respectively.

**Table:**

*D*—H···*A*	*D*—H	H···*A*	*D*···*A*	*D*—H···*A*
O1—H81···N2	0.84	1.87	2.597 (2)	144.
O1—H81···S1	0.84	2.99	3.7096 (14)	145.
O2—H82···N4	0.84	1.89	2.617 (2)	144.
O2—H82···S1	0.84	3.08	3.8135 (16)	147.
N1—H71···S1^i^	0.86 (2)	2.53 (2)	3.3514 (17)	159 (2)
N3—H73···S1^i^	0.85 (3)	2.82 (3)	3.5605 (18)	147 (2)
C16—H16···*C*_g_2^i^	0.95	2.81	3.423 (2)	123
C26—H26···*C*_g_1^i^	0.95	2.74	3.438 (2)	130

Symmetry codes: (i) −*x*+1, *y*+1/2, −*z*+1/2.
